# Comparing Dislodgeable 2,4-D Residues across Athletic Field Turfgrass Species and Time

**DOI:** 10.1371/journal.pone.0168086

**Published:** 2016-12-09

**Authors:** Matthew D. Jeffries, Travis W. Gannon, James T. Brosnan, Gregory K. Breeden

**Affiliations:** 1 Department of Crop and Soil Sciences, North Carolina State University, Raleigh, North Carolina, United States of America; 2 Department of Plant Sciences, University of Tennessee, Knoxville, Tennessee, United States of America; Instituto Agricultura Sostenible, SPAIN

## Abstract

2,4-dimethylamine salt (2,4-D) is an herbicide commonly applied on athletic fields for broadleaf weed control that can dislodge from treated turfgrass. Dislodge potential is affected by numerous factors, including turfgrass canopy conditions. Building on previous research confirming herbicide-turfgrass dynamics can vary widely between species, field research was initiated in 2014 and 2015 in Raleigh, NC, USA to quantify dislodgeable 2,4-D residues from dormant hybrid bermudagrass (*Cynodon dactylon* L. x *C*. *transvaalensis*) and hybrid bermudagrass overseeded with perennial ryegrass (*Lolium perenne* L.), which are common athletic field playing surfaces in subtropical climates. Additionally, dislodgeable 2,4-D was compared at AM (7:00 eastern standard time) and PM (14:00) sample timings within a day. Samples collected from perennial ryegrass consistently resulted in greater 2,4-D dislodgment immediately after application (9.4 to 9.9% of applied) compared to dormant hybrid bermudagrass (2.3 to 2.9%), as well as at all AM compared to PM timings from 1 to 3 d after treatment (DAT; 0.4 to 6.3% compared to 0.1 to 0.8%). Dislodgeable 2,4-D did not differ across turfgrass species at PM sample collections, with ≤ 0.1% of the 2,4-D applied dislodged from 1 to 6 DAT, and 2,4-D detection did not occur at 12 and 24 DAT. In conclusion, dislodgeable 2,4-D from treated turfgrass can vary between species and over short time-scales within a day. This information should be taken into account in human exposure risk assessments, as well as by turfgrass managers and athletic field event coordinators to minimize 2,4-D exposure.

## Introduction

Turfgrasses are used for a variety of societal purposes, including activities on athletic fields. The National Turfgrass Research Initiative reported in 2003 there were > 700,000 managed athletic fields in the US [1]. Maintaining a functional, safe playing surface for participants is the primary objective of athletic field managers, as poor footing conditions can lead to increased lower body injuries [2, 3]. In subtropical climates, warm-season turfgrass species such as bermudagrass (*Cynodon* spp.) are often overseeded during dormancy periods with a cool-season turfgrass to improve athletic field surface aesthetics and functionality [4]. Perennial ryegrass (*Lolium perenne* L.) is a cool-season species commonly utilized for overseeding because of its rapid germination (≈ 5 d), dark-green color and winter hardiness [5]. Regardless of the turfgrass species present on athletic fields, conditions can unfold that degrade playing surface quality and compromise playing surface safety via weed encroachment. Athletic field managers employ multiple weed control practices to alleviate this issue, including synthetic herbicide applications. One such herbicide athletic field managers utilize for selective broadleaf control is 2,4-dimethylamine salt (2,4-D), which is registered for use on cool- and warm-season turfgrasses [6]. Although research to date is inconclusive on human-carcinogenic effects from 2,4-D, it is a confirmed toxin to blood, kidney, and liver, as well as an eye irritant [6–10].

As with any pesticide, various transformation and transport processes ensue following application. Physicochemical properties specific to 2,4-D transport from the intended site include very high water solubility (K_s_ = 796,000 mg L^-1^; 20°C) and a low soil organic carbon sorption coefficient (K_oc_ = 20 mL g^-1^) [6, 11], which suggests 2,4-D may dislodge from treated turfgrass vegetation. Nishioka et al. [12] reported 2,4-D consistently tracked into residences (10 total) from treated residential lawns up to 1 wk following application, with > 70% of total household loading attributed to children’s shoes and dogs. Jeffries et al. [13] reported 2,4-D dislodgment from hybrid bermudagrass fluctuated with daily canopy moisture conditions. More specifically, 4% of the applied was dislodged at 5:00:00 eastern standard time (EST) 1 d after treatment (DAT), which declined to 0.1% by 13:00:00; however, dislodgment increased to 2.1% of the applied at 2 DAT–5:00:00 and similar trends persisted through 6 DAT.

Following pesticide dislodgement from treated turfgrass, various human exposure routes may occur. Specific to 2,4-D, nonoccupational human absorption occurs via dermal, as well as dietary and nondietary ingestion routes [12, 14–16]. Humans do not readily metabolize 2,4-D in the body, and it is ultimately lost via urine. Human exposure to 2,4-D is commonly confirmed via urine sampling, and a notable example of this is the 2001–2002 National Health and Nutrition Examination Survey, which reported urine-2,4-D detection occurrence in over 25% of the 546 children (ages 6 to 11 yr) evaluated [17].

Human pesticide exposure risk assessments are arduous endeavors that estimate the nature and probability of adverse health effects following exposure to contaminated environmental media [18]. Within the occupational and residential exposure test guidelines currently employed by the US Environmental Protection Agency (EPA), foliar dislodgeable residue dissipation tests (OPPTS 875.2100) are required for pesticide registration or re-registration [19]. The purpose of such tests is to quantify pesticide residues remaining on treated surfaces that can be dislodged through various processes on human skin/clothing or inhaled [20]. Within current US EPA protocols, experiment site selection considerations only reference climatic conditions representative of the intended use area [20]. Information pertaining to turfgrass species and management inputs may improve foliar dislodgeable residue dissipation tests, as growth characteristics vary widely between cool- and warm-season turfgrasses, and can affect pesticide-plant interactions [21]. Additionally, herbicide uptake, translocation and metabolism may differ across turfgrasses with relatively comparable growth characteristics (i.e. within cool- or warm-season turfgrasses) [22–24].

The objectives of this research were to quantify dislodgeable 2,4-D foliar residues between turfgrass species over two time scales, both within a day and over days. By doing so, 2,4-D human exposure assessments may be improved through incorporating more site-specific conditions. We hypothesized 2,4-D dislodgeability would vary between species and over the course of both time scales.

## Materials and Methods

### Site Description

Field experiments were initiated 9 April 2014 and 31 March 2015 (Lake Wheeler Turfgrass Field Laboratory, Raleigh, NC, USA; Lat. 35°44’21.34” N, Long. 78°40’49.75” W) on a sandy clay loam soil with pH 6.4 and 1.9% organic matter w w^-1^. Curtis Powell (Lake Wheeler Road Field Laboratory Superintendent, Raleigh, NC, USA) granted permission to access experiment areas. Research was conducted on weed-free areas where 2,4-D had not been applied 2 yr preceding initiation. Prior to experiment initiation, vegetation and soil from research areas were analyzed to confirm nondetectable 2,4-D residues [25].

Research was conducted on established, dormant ‘Tifway’ hybrid bermudagrass alone, as well as areas overseeded (broadcasted at 976 kg pure live seed ha^-1^) with ‘Carly’ perennial ryegrass in the fall prior to experiment initiation. Overseeding occurred on 12 September 2013 and 23 September 2014 for experiments initiated in 2014 and 2015, respectively. All areas were maintained as an athletic field surface with respect to fungicide/insecticide applications, nutrient applications (49 kg N ha^-1^ mo^-1^), irrigation (provided to supplement rainfall) and mowing (1.9 cm height of cut; three events wk^-1^; clippings returned) [4]. Herbicides and plant growth regulators were not applied to experimental areas throughout the research.

### Experimental Design

Research was conducted as a split plot, randomized complete block design with three replicates of a 2-by-2-by-6 factorial treatment arrangement each year. Main plots were split by turfgrass species (dormant hybrid bermudagrass or overseeded perennial ryegrass), with subplots combinations of 2 sample collection times within a day (7:00 or 14:00 EST) in each of 6 sample days (1, 2, 3, 6, 12 or 24 DAT). Dislodgeable residue samples were also collected immediately following application, and after a 1 h drying period on 0 DAT; however, these samples were statistically analyzed separately due to differing collection timings from other samples beyond 0 DAT. A nontreated check was included in all experimental blocks to ensure the trial area was not contaminated.

### Experiment Initiation

One d prior to trial initiation, areas were mown (clippings collected) and irrigated to field capacity. Experimental areas were not irrigated or mown for 6 d following treatment. Furthermore, areas were covered with plastic (HDX 6 Mil Clear Plastic; The Home Depot Corp., Atlanta, GA, USA) suspended above the turfgrass canopy during rainfall events during this 6 d period. At experiment initiation, 2,4-D amine (Amine 400 2,4-D Weed Killer^®^; PBI/Gordon Corp., Kansas City, MO, USA) was applied at 2.1 kg ai ha^-1^ to plots measuring 1.5 by 2.25 m (1 m alleys between blocks to enable sample collection without human-plot contact). 2,4-D was applied in accordance with local regulatory and manufacturer instructions regarding personal protective equipment, sprayer setup and use site. The selected application rate is labeled for use in turfgrass systems (sod production); however, is 20% higher than athletic field allowance. The increased application rate was required to ensure 2,4-D residue detection at 6 DAT, which we felt was a justifiable compromise to better elucidate the research variables of interest. Treatments were sprayed at 14:00:00 to allow the solution to dry on vegetation with ≥ 5 h of sunlight remaining that day. Applications were made with a hand-held CO_2_-pressurized sprayer comprised of four 80015 XR VS flat-fan nozzles (TeeJet^®^ Flat-Fan Nozzles; Spraying Systems Co., Wheaton, IL, USA). The carrier volume selected (187 L ha^-1^ at 179 kPa) is the minimum stated on the label, creating the worst-case scenario for pesticide retention on the turfgrass canopy. To ensure 2,4-D was applied at the intended rate, cellulose-based sheets (387 cm^2^; Whatman™ 3 MM Chr Chromatography Paper, GE Healthcare Bio-Sciences, Pittsburgh, PA, USA) were randomly placed throughout the trial area. Following 2,4-D spray application overtop sheets, residue concentrations were quantified by high performance liquid chromatography (HPLC) with a diode array detector (DAD) analysis. Finally, air temperature, dew point, relative humidity and sunrise were logged throughout experiments (Table 1). Additionally, leaf wetness (Leaf Wetness Sensor; Decagon Devices Inc., Pullman, WA, USA) was measured with a flat-plate sensor facing north at a 45° angle from the ground surface at a 0.6 m height.

**Table 1:** Climatic conditions recorded from 1 to 6 d after treatment^a^^,^ ^b^.

		^___________________________^ 2014 ^___________________________^					^___________________________^ 2015 ^___________________________^				
DAT	TWD^c^	RH	AT	DP	TFS	LW	RH	AT	DP	TFS	LW
		%	°C	°C	min	mV	%	°C	°C	min	mV
1	7:00:00	80	10.3	7.1	10	340	81	2.8	1.7	-3	369
	14:00:00	33	18.7	2.9	430	266	30	20.7	3.1	417	269
2	7:00:00	60	12.8	6.2	12	273	61	10.4	3.2	-1	273
	14:00:00	32	21.4	3.2	432	268	33	16.8	0.9	419	266
3	7:00:00	83	5.6	4.6	13	328	79	5.0	2.9	0	336
	14:00:00	29	24.4	7.2	433	265	34	20.9	5.6	420	271
6	7:00:00	80	16.5	13.6	17	278	65	3.7	-1.2	4	272
	14:00:00	52	13.6	13.6	437	274	23	17.7	-3.4	424	267

^a^ Abbreviations: DAT, d after treatment; TWD, time within a d; RH, relative humidity; AT, air temperature; DP, dew point; TFS, time from sunrise; LW, leaf wetness.

^b^ Climatic conditions recorded on site at the Lake Wheeler Turfgrass Field Laboratory (Raleigh, NC, USA).

^c^ Eastern standard time.

### Sample Collection

#### Dislodgeable 2,4-D

**Fig 1:**
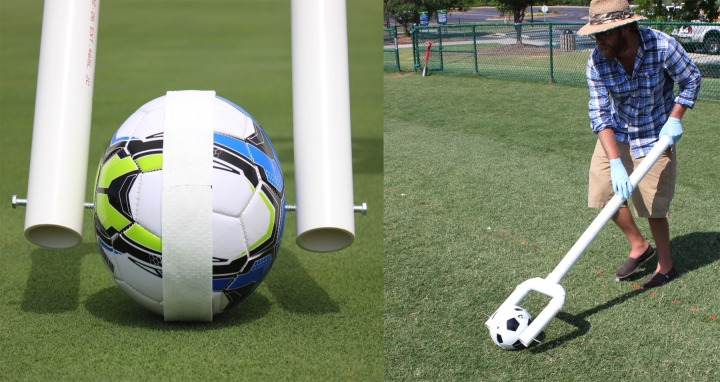
Soccer Ball Roller Apparatus for Dislodge Sampling. Frame constructed of PVC (5 cm inner diameter) and lag bolts to mount a soccer ball, which allows for a consistent end-over-end ball roll and constant sorbent strip-to-turfgrass contact.

Dislodgeable 2,4-D was quantified by rolling a soccer ball (Size 4; Franklin Sports Competition 100 Soccer Ball, Franklin Sports, Stoughton, MA, USA) over a 9 m distance (four 2.25 m side-by-side rolls) within each sub-sub plot. 2,4-D dislodgment via this process was selected due to soccer’s rank as the most popular international sport coupled with the frequency of ball-to-turfgrass and subsequent ball-to-hand contacts inherent to play [26]. Ball roll distance was based off of a soccer ball rolling 50% of the recommended field length (18 m) for youth ages < 6 yr in the US [27]. The soccer ball was double-wrapped with a 5 by 120 cm cellulose-based sorbent strip (Scott Shop Towel™; Kimberly-Clark Corp., Neenah, WI, USA). The soccer ball was mounted to a hand-held PVC apparatus designed such that the ball rotated end-over-end in the same direction as the sorbent strip, thus allowing for constant sorbent strip contact to the treated turfgrass surface (Fig 1; the individual in this figure has given written informed consent outlined in the PLOS consent form to publish these details). While this is not representative of a ball roll when actively playing soccer, this measure was required to minimize variation in data and determine the maximum dislodgeable 2,4-D from turfgrass vegetation. Following ball roll, the entire sorbent strip was removed, placed in a unique glass jar (473 cm^3^) and stored at -12°C for subsequent extraction and HPLC-DAD analysis. Dislodgeable 2,4-D residues relative to the amount applied at trial initiation was calculated using the equation:

$$\%\;\mathrm{dislodged}\;\mathrm{of}\;\mathrm{applied}=\left[\left(\mathrm{BR}\;\mu g\;{2,4\text{-}D\;\mathrm{cm}}^{-2}÷20.9\;\mu g\;{2,4\text{-}D\;\mathrm{cm}}^{-2}\right)\times 100\right]$$ (1)

where BR represents 2,4-D residue recovered from ball roll samples relative to the 2,4-D application rate (20.9 μg 2,4-D cm^-2^).

#### Turfgrass Vegetation Residues

The quantity of 2,4-D within or on the surface of turfgrass vegetation (i.e., in/on) was quantified at all sample collections to: 1) quantify vegetation spray interception across species; and 2) characterize 2,4-D dissipation in/on dormant hybrid bermudagrass and perennial ryegrass vegetation over time. This was done by collecting a core (10.8 cm diam; 92 cm^2^) such that sampling equipment did not contact aboveground vegetation. Following collection, all samples were frozen, turfgrass vegetation was harvested, weighed, processed [1.7 mm (Fitzmill Homoloid Model JT 6; Fitzpatrick Co., Elmhurst, IL, USA)] and stored at -12°C until extraction and residue analysis. 2,4-D residue in/on turfgrass vegetation was calculated as a percent of the amount applied at trial initiation was calculated with the equation:

$$\%\;\mathrm{of}\;\mathrm{applied}=\left[\left(\mathrm{AV}\;\mu g\;{2,4\text{-}D\;\mathrm{cm}}^{-2}÷20.9\;\mu g\;{2,4\text{-}D\;\mathrm{cm}}^{-2}\right)\times 100\right]$$ (2)

where AV represents 2,4-D residue recovered from aboveground vegetation as a percent of the 2,4-D application rate (20.9 μg cm^-2^).

### Residue Analyses

2,4-D residues were quantified with HPLC-DAD (Agilent-1260 Infinity; Agilent Technologies, Inc., Wilmington, DE, USA) methodology. Details pertaining to sample preparation, extraction and cleanup, as well as analytical parameters are provided in Jeffries et al. [13]. In summary, limits of detection and quantification were 0.3 and 1.0 mg L^-1^, respectively, while maintaining the signal to noise ratio at 3:1. 2,4-D residue concentrations were quantified using peak area measurements (OpenLAB CDS ChemStation, Version C.01.04; Agilent Technologies, Inc., Wilmington, DE, USA) and concentrations above the calibration curve were diluted and re-injected for analysis. Fortification recovery checks for sorbent strips and vegetation matrices ranged from 93 to 103 and 90 to 96%, respectively, across all analyses conducted in the presented research. Lastly, application recovery check sheets determined 2,4-D was applied at 95% of the intended rate across years.

### Statistical Analyses

Statistical analyses were conducted by ANOVA (P = 0.05) using MIXED procedures in SAS (Statistical Analysis Software^®^, Version 9.2; SAS Institute, Inc., Cary, NC, USA). Turfgrass species and sample collection timings were considered fixed effects, while year and replicate were considered random as described by Carmer et al. [28]. Main effects and their interactions are presented accordingly, with precedent given to significant interactions of increasing magnitude [29]. Means were separated according to Fisher’s protected LSD (P < 0.05) and Pearson correlation coefficients (P = 0.05) were determined to quantify the relationships between selected climatic conditions with dislodgeable 2,4-D plant residues.

## Results and Discussion

### 2,4-D Dislodgeability

2,4-D residue detection did not occur in dislodgment samples collected 12 and 24 DAT; therefore, these data were excluded from statistical analysis. Nondetection at 12 and 24 DAT may be due in part to irrigation or precipitation inputs following 6 DAT sample collection, as previous research has shown dislodgment of the highly water soluble herbicide is reduced following water inputs [13, 30]. ANOVA revealed significant interactions for all sources of variation including year; therefore, data were sorted by year and presented accordingly. Additionally, ANOVA revealed a significant turfgrass-by-sample collection time within a day interaction from 0 to 6 DAT, which is presented. Across years, greater 2,4-D dislodgment occurred from perennial ryegrass compared to dormant hybrid bermudagrass. Within perennial ryegrass, greater 2,4-D dislodgment occurred at AM compared to PM sample collections. Samples collected immediately after application resulted in > three-fold greater dislodgment in perennial ryegrass (9.4 to 9.9% of applied across runs) compared to dormant hybrid bermudagrass (2.3 to 2.9%), while a 1 h dry time decreased dislodge to ≤ 0.5% across species (Table 2). At 1 DAT, 3.7 and 6.3% of the applied 2,4-D dislodged from perennial ryegrass in 2014 and 2015, respectively, while less dislodged from dormant hybrid bermudagrass (0.6 to 0.8%). Across species in 2014, greater 2,4-D dislodgment occurred at 1 DAT in AM (0.6 to 3.7% of applied) compared to PM (0.1%) sample collections, while this was only true for perennial ryegrass in 2015 (6.3 and 0.1% of applied in AM and PM, respectively). At 2 and 3 DAT across years, greater 2,4-D dislodgment occurred from perennial ryegrass than dormant hybrid bermudagrass in the AM (0.4 to 4.1 and ≤ 0.6% of applied, respectively). Additionally, 2,4-D dislodgment declined to ≤ 0.1% of the applied in the PM across turfgrass species. Although statistical separation is not permissible at 6 DAT, 2,4-D detection occurred for both turfgrass species and sample collection times in 2014 (≤ 1.7% of applied), and only for perennial ryegrass–AM in 2015 (0.2%).

**Table 2:** Dislodgeable 2,4-D residues from dormant hybrid bermudagrass (*C*. *dactylon* x *C*. *transvaalensis*) and overseeded perennial ryegrass (*Lolium perenne*) at morning and afternoon sampling times^a^^,^ ^b^.

	^__________________________________________________^ 2014 ^__________________________________________________^									
	^____^ 0 DAT ^_____^		^____^ 1 DAT ^____^		^____^ 2 DAT ^____^		^____^ 3 DAT ^____^		^____^ 6 DAT ^____^	
Turfgrass	0 h^c^	1 h	AM	PM	AM	PM	AM	PM	AM	PM
	^_______________________________________^ % dislodged of applied ^_______________________________________^									
Hybrid bermudagrass	2.3	0.1	0.6	0.1	< 0.1	< 0.1	0.1	ND	0.3	< 0.1
Perennial ryegrass	9.9	0.5	3.7	0.1	1.1	0.1	2.2	0.1	1.7	< 0.1
LSD_0.05_^d^	^______^ 2.9 ^______^		^______^ 0.5 ^______^		^______^ 0.4 ^______^		^______^ 0.4 ^______^		^______^ NS ^______^	
	^__________________________________________________^ 2015 ^__________________________________________________^									
	^____^ 0 DAT ^_____^		^____^ 1 DAT ^____^		^____^ 2 DAT ^____^		^____^ 3 DAT ^____^		^____^ 6 DAT ^____^	
Turfgrass	0 h	1 h	AM	PM	AM	PM	AM	PM	AM	PM
	^_______________________________________^ % dislodged of applied ^_______________________________________^									
Hybrid bermudagrass	2.9	< 0.1	0.8	< 0.1	ND	< 0.1	0.6	< 0.1	ND	ND
Perennial ryegrass	9.4	0.2	6.3	0.1	0.4	0.1	4.1	< 0.1	0.2	ND
LSD_0.05_^d^	^______^ 2.9 ^______^		^______^ 0.3 ^______^		^______^ 0.1 ^______^		^______^ 1.1 ^______^		^______^ NS ^______^	

^a^ Abbreviations: DAT, d after treatment; AM, 7:00:00 EST; PM, 14:00:00 EST; ND, nondetection; NS, nonsignificant.

^b^ Dislodgeable 2,4-D residue detection did not occur at 12 and 24 DAT.

^c^ Dislodge samples collected immediately and 1 h following application.

^d^ LSD for comparing turfgrass by sample time within a d interaction.

**Fig 2:**
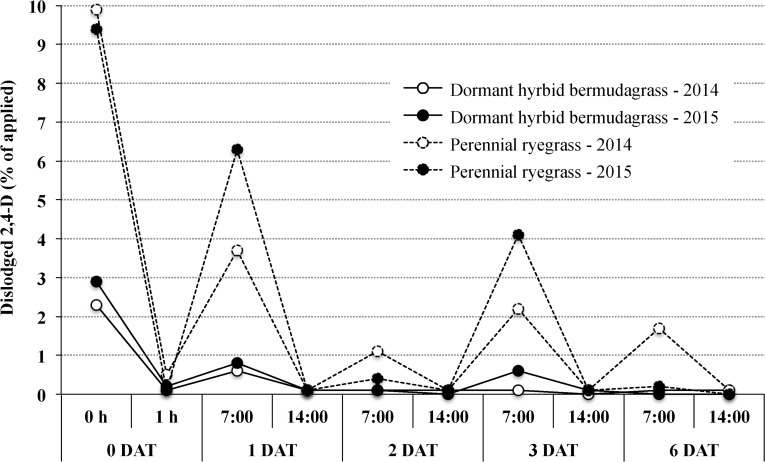
Dislodgeable 2,4-D Residues from Dormant Hybrid Bermudagrass and Actively Growing Perennial Ryegrass Over Time. Dislodged 2,4-D following one soccer ball roll (0.19 m^2^) over turfgrass as a percent of the application rate (2.1 kg ai ha^-1^). Sample collections occurred at 0 h (14:00) and 1 h (15:00) after application on 0 d after treatment (DAT), and 7:00 and 14:00 from at AM and PM sample collections, respectively.

Although statistical separation is not permissible as presented, dislodged 2,4-D from perennial ryegrass consistently increased from PM sampling on a given day to AM sampling the subsequent day (Fig 2.). This trend also occurred in dormant hybrid bermudagrass from 0 DAT–1 h to 1 DAT–AM, and sporadically through 6 DAT. This may be due to 2,4-D re-suspension overnight on turfgrass vegetation as conditions become more favorable for moisture development. Jeffries et al. [13] reported similar trends from actively growing hybrid bermudagrass, as 2,4-D dislodged 1 DAT–13:00 (0.1% of applied) increased at 2 DAT–5:00 (2.1%)

### 2,4-D Persistence in Turfgrass Vegetation

ANOVA revealed significant interactions for all sources of variation including year; therefore, data were sorted by year. 2,4-D persistence varied between species at various DAT, and did not vary between sample collections in each day within species; therefore, the main effect of turfgrass species is presented and discussion is focused on highlighting factors that may have contributed to varying dislodgeable 2,4-D results between perennial ryegrass and dormant hybrid bermudagrass. When statistical separation occurred between turfgrass species, 2,4-D persistence in perennial ryegrass turfgrass vegetation outranked dormant hybrid bermudagrass. Although not statistically different in 2014, samples collected at 0 DAT suggest 2,4-D spray interception varied between species, as 75 to 96% of the applied was recovered in perennial ryegrass vegetation compared to 52 to 82% in hybrid bermudagrass (Table 3). This may be due in part to morphological differences between actively growing perennial ryegrass and dormant hybrid bermudagrass, which this study is unable to confirm. Additionally, this may be due in part to increased aboveground biomass in a hybrid dormant bermudagrass area overseeded with perennial ryegrass compared to solely a dormant hybrid bermudagrass area. Averaged over years, fresh aboveground biomass core^-1^ was 4.9 and 9.3 g from dormant hybrid bermudagrass and perennial ryegrass, respectively (data not shown). In general, greater vegetation-spray interception occurred in 2015, which may also be attributed to greater aboveground biomass between years. Dormant hybrid bermudagrass and perennial ryegrass fresh aboveground biomass core^-1^ was 206 and 150% greater in 2015 than 2014, respectively (data not shown).

**Table 3:** 2,4-D residue persistence in dormant hybrid bermudagrass (*C*. *dactylon* x *C*. *transvaalensis*) and overseeded perennial ryegrass (*Lolium perenne*) aboveground vegetation^a^^,^ ^b^.

	^__________________________________________________^ 2014 ^__________________________________________________^						
Turfgrass	0 DAT	1 DAT	2 DAT	3 DAT	6 DAT	12 DAT	24 DAT
	^_____________________________________________^ % of applied ^_____________________________________________^						
Hybrid bermudagrass	52	54	50	38	39	3	1
Perennial ryegrass	75	69	68	60	55	7	2
LSD_0.05_^c^	NS	NS	17	NS	NS	2	NS
	^__________________________________________________^ 2015 ^__________________________________________________^						
Turfgrass	0 DAT	1 DAT	2 DAT	3 DAT	6 DAT	12 DAT	24 DAT
	^_____________________________________________^ % of applied ^_____________________________________________^						
Hybrid bermudagrass	82	61	63	62	47	3	1
Perennial ryegrass	96	83	86	76	51	5	2
LSD_0.05_^c^	11	14	NS	7	NS	1	1

^a^ Abbreviations: DAT, d after treatment; NS, nonsignificant.

^b^ Irrigation/precipitation and mowing did not occur from 0 to 6 DAT sample collection.

^c^ LSD for comparing turfgrasses within a d after application.

Relative to respective 0 DAT data, 2,4-D persisted similarly in/on turfgrass vegetation across species, as persistence declined 25 and 27% in dormant hybrid bermudagrass and perennial ryegrass in 2014 and 43 and 47% in 2015, respectively, through 6 DAT. Similar dissipation rates between dormant hybrid bermudagrass and actively growing perennial ryegrass were not expected, and the presented research cannot elucidate the reason for this occurrence; however, the amine 2,4-D formulation evaluated in this research has poor foliar uptake compared to other formulations, which application and management practices through 6 DAT favored [31]. Additionally, Weintraub et al. [32] reported 2,4-D was metabolized in dormant cherry tree (*Prunus avium*) buds, as > 90% ^14^C-2,4-D dissipation occurred over 3 to 5 mo dormancy periods. The dramatic 2,4-D residue decline in turfgrass vegetation from 6 to 12 DAT is likely due to irrigation or precipitation inputs coupled with mowing events during this time period. Finally, 2,4-D detection in/on turfgrass vegetation occurred consistently across years and turfgrasses at 24 DAT, suggesting management practices to minimize off-target transport should be employed for at least this duration following application.

### Climatic Condition Correlations with Dislodgeable 2,4-D

Climatic data from 0 to 6 DAT suggest dislodgeable 2,4-D may be influenced by conditions favoring turfgrass canopy moisture. Relative humidity is a measurement of atmospheric moisture relative to saturated air, with increasing values suggesting increased atmospheric moisture [33]. Dew point is the air temperature below which moisture in the atmosphere condenses, and as difference in relative humidity and dew point decrease, dew formation becomes more likely [13, 34]. Maximum dew formation on plant canopies has previously been reported to occur at, or just after sunrise [13, 35, 36]. Although turfgrass canopy moisture is not solely influenced by these climatic parameters, previous research has shown they are correlated with 2,4-D dislodge from treated vegetation [13]. Jeffries et al. [13] reported correlations between dislodgeable 2,4-D at 1 DAT and air temperature–dew point, leaf wetness, relative humidity and time from sunrise were -0.73 (P < 0.0001), 0.58 (P < 0.01), 0.69 (P < 0.0001) and -0.82 (P < 0.0001), respectively.

Pooled over data from 1 to 6 DAT, 2,4-D dislodgeability from dormant hybrid bermudagrass was strongly correlated (*r* ≥ 0.7) with leaf wetness in both years (*r* = 0.85 to 0.94) and relative humidity in 2015 (*r* = 0.73), suggesting dislodgment increased as leaf wetness and atmospheric moisture increased (Table 4). These variables were also strongly correlated with 2,4-D dislodgment from perennial ryegrass (leaf wetness *r* = 0.82 to 0.96; relative humidity *r* = 0.71 to 0.8). Additionally, strong negative correlations were detected between 2,4-D dislodgment from perennial ryegrass and air temperature–dew point in both years (*r* = -0.77 to -0.79) and time from sunrise in 2014 (*r* = -0.81), suggesting dislodgment increased as air temperature approached dew point and time from sunrise decreased.

**Table 4:** Pearson correlation coefficients quantifying the relationships between climatic parameters and dislodgeable 2,4-D residues from dormant hybrid bermudagrass (*C*. *dactylon* x *C*. *transvaalensis*) and overseeded perennial ryegrass (*Lolium perenne*)^a^^-^^c^.

	^___________________^ % 2,4-D dislodged of applied ^___________________^			
	^______________^ 2014 ^______________^		^______________^ 2015 ^______________^	
Climatic parameter	BER	RYE	BER	RYE
	^____________________________________^ *r* ^____________________________________^			
Air temp–dew point	-0.38	-0.79***^d^	-0.69***	-0.77†
Leaf wetness	0.85†	0.82†	0.94†	0.96†
Relative humidity	0.42*	0.71†	0.73†	0.80†
Time from sunrise	-0.40	-0.81†	-0.53**	-0.69**

^a^ Abbreviations: BER, hybrid bermudagrass; RYE, perennial ryegrass.

^b^ Climatic conditions recorded on site at the Lake Wheeler Turfgrass Field Laboratory

(Raleigh, NC, USA).

^c^ Data pooled over 1 through 6 d after treatment sample collections.

^d^ †, ***, ** and * denote significance at P < 0.0001, 0.001, 0.01 and 0.05, respectively.

Specific to perennial ryegrass, dislodgeable 2,4-D at AM samplings declined from 1 to 2 DAT, and increased from 2 to 3 DAT in both years, which may be explained in part by climatic conditions. More specifically, 2 DAT–AM had lower relative humidity (60 and 61%), the largest difference between air temperature and dew point (6.6 and 7.2°C) and lowest leaf wetness counts (273 mV) than other AM sample collections from 1 to 6 DAT (excluding 2015–6 DAT; Table 1). Wichink Kruit et al. [37] reported a 71% relative humidity threshold was required for moisture development on grass canopies, which likely varies based on site-specific conditions; however, increased relative humidity generally aligned with increasing leaf wetness measurements through 3 DAT in our research.

### Research Implications

Data from the presented research were used to predict daily human dermal 2,4-D exposure as calculated by US EPA for post-application exposure for physical activities on turfgrass using the equation:

$$E={\mathrm{TTR}}_{t}\times \mathrm{CF}\times \mathrm{TC}\times \mathrm{ET}$$ (3)

where E = exposure (mg d^-1^); TTR_t_ = turf transferable residue on day t (X μg 2,4-D cm^2^); CF = unit conversion factor (0.001 mg μg^-1^); TC = transfer coefficient (49,000 cm^2^ hr^-1^; 1 to 2 yr children); and ET = exposure time (1.5 hr d^-1^; 1 to 2 yr children) in the algorithm [13, 38]. The algorithm, as well as TC and ET coefficients were obtained from the US EPA risk assessment, while TTR_1_ (X μg 2,4-D cm^2^) corresponds to the dislodgment data in the presented research [38]. Comparing the highest dislodgeable residue values from each turfgrass species after the day of application (2015–1 DAT–AM), it was calculated that a human could potentially be exposed to 12 and 96 mg 2,4-D d^-1^ from one soccer ball roll over a 0.19 m^2^ area of dormant hybrid bermudagrass and perennial ryegrass, respectively. Adjusting this for the 10% 2,4-D dermal absorption rate utilized in the US EPA risk assessment results in 1.2 and 9.6 mg 2,4-D d^-1^ from dormant hybrid bermudagrass and perennial ryegrass, respectively [6, 13, 38]. Using the risk assessment value for short-term (30 d) human dermal exposure of 25 mg kg^-1^ d^-1^, it was determined that an average 1 to 2 yr old child (11 kg) may be dermally exposed to 275 mg 2,4-D d^-1^ without adverse effect [6, 13]. By calculating our observed maximum daily exposure (1.2 and 9.6 mg 2,4-D d^-1^) as a percent of the short-term daily dermal exposure allowance without adverse effect (275 mg 2,4-D d^-1^), it was determined that 2,4-D dislodged from one ball roll equaled 0.4 and 3.5% of this limit from dormant hybrid bermudagrass and perennial ryegrass, respectively. Across turfgrass species, all PM sample collections from 1 to 6 DAT resulted in ≤ 0.06% of the daily limit. It should be noted again that the 2,4-D application rate in the presented research was 20% greater than current label allowances on athletic fields, which overestimates in situ exposure with this algorithm; however, the area covered by one ball roll in this research equals 0.07% of the area of the smallest children’s (U6) soccer field (14 by 18 m) recommended by the US Youth Soccer Organization [13, 27].

The presented research is not intended to detract from the approach current regulatory agencies use to estimate human 2,4-D exposure. Instead, the intention is to provide information to improve such efforts. To our knowledge, previous research efforts have not compared 2,4-D dislodgment between turfgrass species simultaneously, and excluding Jeffries et al. [13], reports have either not specifically stated when sample collections occurred within a day or were collected after 10:00:00 EST [30, 39, 40]. For example, the sole field experiment evaluating human 2,4-D dermal exposure from treated turfgrass in the 2005 re-registration package collected samples at 12:30:00 EST (0 and 1 DAT) [6, 30]. Results from this research suggest 2,4-D human exposure assessments may be improved by including additional sample collections at earlier and later times of day when canopy moisture is more likely to be present. Additionally, the aforementioned experiment does not specifically state the turfgrass species in the report, which this research confirms is an influencing factor on 2,4-D dislodgeability [30].

## Conclusions

This research built on preceding efforts evaluating dislodgeable 2,4-D from treated turfgrass. Our experiment quantified dislodgeable 2,4-D from two common athletic field turfgrass species via soccer ball roll, a process common to the most popular international sport. Results indicate 2,4-D can dislodge from dormant hybrid bermudagrass and perennial ryegrass up to 6 DAT when water inputs and mowing practices are not employed following application. Ultimately, 2,4-D’s physicochemical properties coupled with varying canopy dynamics between turfgrass species resulted in differing dislodgment between species and sample collection times within a day. More specifically, perennial ryegrass possessed more aboveground biomass, which resulted in greater 2,4-D spray-vegetation interception. Coupling this with increased morning canopy moisture at samplings and 2,4-D’s very high water solubility resulted in maximum 2,4-D dislodgment with perennial ryegrass–AM samplings. Excluding the day of application, ≤ 0.1% of the applied 2,4-D was dislodged at PM samplings across species, suggesting human activity on recently treated fields is safe when canopy moisture is not present.

In conclusion, dislodgeable 2,4-D on athletic fields can vary depending on turfgrass canopy characteristics, and information pertaining to species and conditions favoring canopy moisture presence should be included in human exposure risk assessments. Based off 2,4-D dislodgeable residues measured via soccer ball roll in this research, a relatively nonaggressive approach compared to other athletic processes, these data suggest turfgrass managers and athletic field event schedulers should coordinate 2,4-D application to avoid human activity when canopy moisture is present for at least 6 d following treatment; however, this period will likely vary depending on site-specific conditions and management practices. Future research should investigate similar research objectives with additional pesticides, dislodge methods and turfgrass species, as well as the effect of sprayer setup, surfactant tank-mixes and mowing practices to reduce dislodgeable 2,4-D residues from turfgrass.
